# Trends in Point of Care Ultrasound Familiarity Among Undergraduate Medical Clerkship Educators

**DOI:** 10.24908/pocus.v9i1.16678

**Published:** 2024-04-22

**Authors:** Nilan Schnure, Mahmoud Mohamed Elfadil, Wilma Chan, Cameron Baston, Frances Shofer, Nova Panebianco

**Affiliations:** 1 Section of Hospital Medicine, University of Pennsylvania Philadelphia, PA USA; 2 Division of Emergency Ultrasound, Department of Emergency Medicine, University of Pennsylvania Philadelphia, PA USA; 3 Department of Medicine, Perelman School of Medicine, University of Pennsylvania Philadelphia, PA USA

**Keywords:** POCUS, Point of care ultrasound, bedside ultrasonography, ultrasound training, ultrasound education, medical education, undergraduate clerkship

## Abstract

Objectives: Despite growing use of point of care ultrasound (POCUS), there remains a paucity of data about familiarity with POCUS among educators who dictate curricular content in undergraduate medical education. This paper aims to longitudinally characterize the level of comfort and frequency of POCUS use among faculty involved in undergraduate clerkship education. Methods: A web-based cross-sectional survey assessing comfort, frequency of use, and awareness of indications for POCUS among faculty involved in Internal Medicine, Family Medicine, and Surgery undergraduate clerkship education in a single urban academic medical center in 2016 and again in 2022. Results: A total of 45 responses from 2016 and 30 responses from 2022 are included. The percentage of faculty “not comfortable” with performing POCUS decreased from 78% to 46%, although the overall change in comfort was not statistically significant. Comfort interpreting POCUS images, frequency of POCUS use, and familiarity with the clinical applications of POCUS all improved. Faculty identified multiple barriers to more frequent POCUS use. Conclusions: Over a six-year period at one urban, academic medical center, comfort with POCUS and frequency of use have increased slightly but remain low among core faculty responsible for clerkship education. There are still large gaps in knowledge and very few faculty regularly use POCUS, which can be attributed to multiple different barriers.

## Introduction

Point of care ultrasound (POCUS) instruction in undergraduate medical education is widespread but variable. In a 2019 survey, 73% of United States accredited medical schools indicated having a POCUS curriculum, 84% of which were mandatory 1. POCUS was primarily taught by Emergency Medicine physicians and was often integrated into specific basic science or clinical skills courses, with 35% of instruction occurring on clinical rotations. 

As noted in a recent international consensus statement 2, an ideal POCUS curriculum can enhance the learning of clinical sciences through the integration of POCUS into clinical problem solving and through the care of patients at their point of care. Yet, the rapid inclusion of POCUS into undergraduate medical education is challenged because medical students’ knowledge on this subject may exceed that of supervising physicians who did not receive this training. Lack of attending physician awareness or comfort with POCUS can create a barrier for medical students and resident trainees who wish to use or expand their skill set. This knowledge deficit may be particularly impactful when the untrained supervisor is responsible for establishing learning goals and experiences for trainees.

The increased utilization of POCUS has been recognized, 3, 4, 5, 6 especially in Emergency Medicine^7^. Nevertheless, there is not enough investigation on the level of experience with POCUS among educators of medical students and how this has changed over time. The aim of this paper is to describe the level of comfort with POCUS, frequency of POCUS use, and knowledge of indications for POCUS among faculty involved in the undergraduate clerkship education at a single urban academic medical center using surveys from both 2016 and 2022.

## Materials and Methods

### Study Design 

We conducted a web-based cross-sectional survey assessing comfort, frequency of use, and awareness of indications for POCUS among faculty involved in undergraduate clerkship education in a single urban academic medical center in both 2016 and in 2022. The University of Pennsylvania Institutional Review Board (IRB) exempted the study protocol from full IRB review in both iterations.

### Population and Setting

The target survey population included faculty who were responsible for the curriculum or clinical teaching on medical student clerkship rotations. These included clerkship directors, clinical site directors, and core clinical preceptors. In 2016, this survey was distributed to faculty involved in the Internal Medicine, Family Medicine, General Surgery, Emergency Medicine, and Pediatric Emergency Medicine clerkships, as identified by the School of Medicine’s Curriculum Office. In 2022, a repeat survey was distributed to faculty in similar positions, although the distribution method differed. In 2016, invitations were distributed through a dean’s email distribution list for core faculty, while in 2022, due to a change in medical school leadership, invitations were distributed directly by clerkship directors to faculty in specified roles. While clinical educators from Emergency Medicine and Pediatric Emergency Medicine participated in the 2016, very few responses were received from these specialties in 2022; therefore, this paper reports results from only Internal Medicine, Family Medicine, and Surgery. Subset analyses were also performed for primary care specialties (Internal Medicine and Family Medicine) and paired responses across the two timepoints.

This set of roles was chosen because these educators dictate the curricular content, clinical experiences, and educational priorities for medical students during their core rotations of the clerkship year. In this paper we analyze responses from faculty focusing on the trends of change between two different time periods. 

### Survey Content and Administration

The survey examined respondents’ demographics, comfort with acquiring and interpreting POCUS images, personal frequency of POCUS use, and knowledge of possible indications for POCUS. Comfort was assessed on a four-point scale including “not comfortable,” “somewhat comfortable,” “moderately comfortable,” “extremely comfortable.” To assess knowledge regarding the role of POCUS, respondents were presented with brief clinical vignettes and explicit suspected diagnoses. They were then asked whether POCUS could be used by a trained provider in a theoretical diagnostic evaluation. Scenarios included 12 possible diagnoses for which POCUS is generally considered helpful in the diagnostic workup, as well as three diagnoses for which POCUS is not commonly used (stroke, urinary tract infection, and acute otitis media). Vignettes included a range of pediatric, adult, medical, and surgical pathologies. They were developed by POCUS content experts and reflected both common and uncommon POCUS applications, falling within published guidelines regarding core emergency medicine POCUS applications 8. These survey questions were pilot tested among emergency medicine faculty members prior to distribution.

The survey content was designed to assess the respondent's knowledge regarding the utility of POCUS exams for commonly faced clinical problems, regardless of the ability of the respondents themselves to perform or interpret the exam. This survey design was able to assess general knowledge regarding POCUS that experts expect from medical students’ educators.

The survey was conducted through Research Electronic Data Capture (REDCap®, Nashville, TN), a secure web application for building and managing online surveys and databases. Respondents were sent an invitation to participate via email, with two subsequent email reminders. In the first iteration these were distributed between December 2015 and February 2016, and were sent by the Senior Vice Dean for Education. In the second iteration, invitations were sent between April and May 2022, and were distributed by the clerkship director for each respective specialty. Participants were asked to enter a semi-unique identifying code consisting of the first three letters of their birth city and the two digits of their birthday, which allowed for anonymized responses that could be linked across time points. For presenting the results throughout the manuscript, we refer to the first timepoint as 2016 and the second timepoint as 2022.

### Statistical Analysis

Descriptive and contingency statistical analyses were performed. Results are presented in frequencies. Parametric variables are presented as means ± standard deviation. Non-parametric variables are presented in median and interquartile ranges (IQR). Two-tail Fisher’s exact test was used to examine for contingency for trends of variables between the study timepoints. Two-tail Student’s t-Test was used to examine for difference in means for continuous variables.

In testing for knowledge regarding the role of POCUS in specific clinical diagnoses, we combined ratings of “never” and “sometimes” together as responses reflective of no or minimal role for POCUS. We combined ratings of “often” and “always” together as responses reflective of a role for POCUS. 

## Results

### Characteristics of Study Participants

In 2016, a total of 88 of 192 surveys were completed (46% response rate). Of those completed, 45 were included in the analysis, and came from Internal Medicine (22, 48.8%), Family Medicine (16, 35.6%), or Surgery (7, 15.6%). The median length of years after residency for the 2016 clinical educators cohort was 21 years (IQR 10—31). Educational roles included 9 (20.0%) clerkship directors, 15 (33.3%) site directors, 38 (84.4%) clinical educators in the ward, clinic, or operating room, and 16 (35.6%) didactic educators.

In 2022 a total of 30 of 97 surveys were completed (31% response rate), of which 28 came from either Internal Medicine (8, 28.6%), Family Medicine (14, 50.0%), or Surgery (6, 21.4%). The median length of years after residency for the 2022 clinical educators cohort was 15 years (IQR 5—25). Participants included 5 (17.9%) clerkship directors, 10 (35.7%) site directors, 23 (82.1%) clinical educators in the ward, clinic, or operating room, and 10 (35.7%) didactic educators. Years since training and current educational roles are summarized in Table 1.

**Table 1:** Demographics of clerkship faculty survey respondents: 2016 and 2022 cohorts.

**Variables**	**2016** **Respondents** **(n = 45)**	**2022** **Respondents (n=28)**
Specialty, n (%) · Internal medicine · Surgery · Family medicine	22 (48.8) 7 (15.6) 16 (35.6)	8 (28.6) 6 (21.4) 14 (50)
Educational Role, n (%) · Clerkship director · Site director · Clinical teaching · Didactic teaching	9 (20) 15 (33.3) 38 (84.4) 16 (35.6)	5 (17.9) 10 (35.7) 23 (82.1) 10 (35.7)
Years since completing residency, years, median (IQR)	21 (10—31)	15 (5—25)
IQR = Interquartile ranges		

### Comfort and Frequency of POCUS Use

Respondents were asked how comfortable they were performing POCUS examinations, as well as interpreting the POCUS images performed either by themselves or another individual (Table 2). Comfort performing POCUS improved from 2016 to 2022, although this was not statistically significant (p=0.053). Those who responded that they were not comfortable performing POCUS decreased from 77.8% to 46.4%. Moreover, those who responded that they were moderately or extremely comfortable performing POCUS increased from 11.1% and 2.2% in 2016 to 28.6% and 7.1% in 2022, respectively.

Regarding interpretation of POCUS, those who were not comfortable interpreting scans decreased from 75.6% in 2016 to 46.4% in 2022. Additionally, those who were moderately comfortable interpreting scans increased from 8.9% in 2016 to 25% in 2022, and those who were extremely comfortable interpreting scans increased from 0% in 2016 to 7.1% in 2022 (p=0.032). Those who used POCUS several times a month increased from 6.7% in 2016 to 14.3% in 2022, and those who used POCUS several times a week increased from 2.2% to 10.7% (p=0.004).

**Table 2:** Comfort in performing and interpreting POCUS images and personal frequency of POCUS use in 2016 and 2022 cohorts.

**Variables**, n (%)	**Likert Scale **				**P-Value**
	**Not comfortable**	**Somewhat comfortable**	**Moderately comfortable**	**Extremely comfortable**	
Comfort performing POCUS: · 2016 (n=45) · 2022 (n=28)	35 (77.8) 13 (46.4)	4 (8.9) 5 (17.9)	5 (11.1) 8 (28.6)	1 (2.2) 2 (7.1)	0.053
Comfort interpreting POCUS images: · 2016 (n=45) · 2022 (n=28)	34 (75.6) 13 (46.4)	7 (15.6) 6 (21.4)	4 (8.9) 7 (25)	0 (0) 2 (7.1)	0.032
	**Never**	**Rarely** (Once or twice per month)	**Sometimes** (several times per month)	**Frequently** (several times per week)	
Frequency of use of POCUS: · 2016 (n=45) · 2022 (n=28)	35 (77.8) 10 (35.7)	6 (13.3) 11 (39.3)	3 (6.7) 4 (14.3)	1 (2.2) 3 (10.7)	0.004
POCUS = Point of care ultrasound.					

### Knowledge

**Figure 1:**
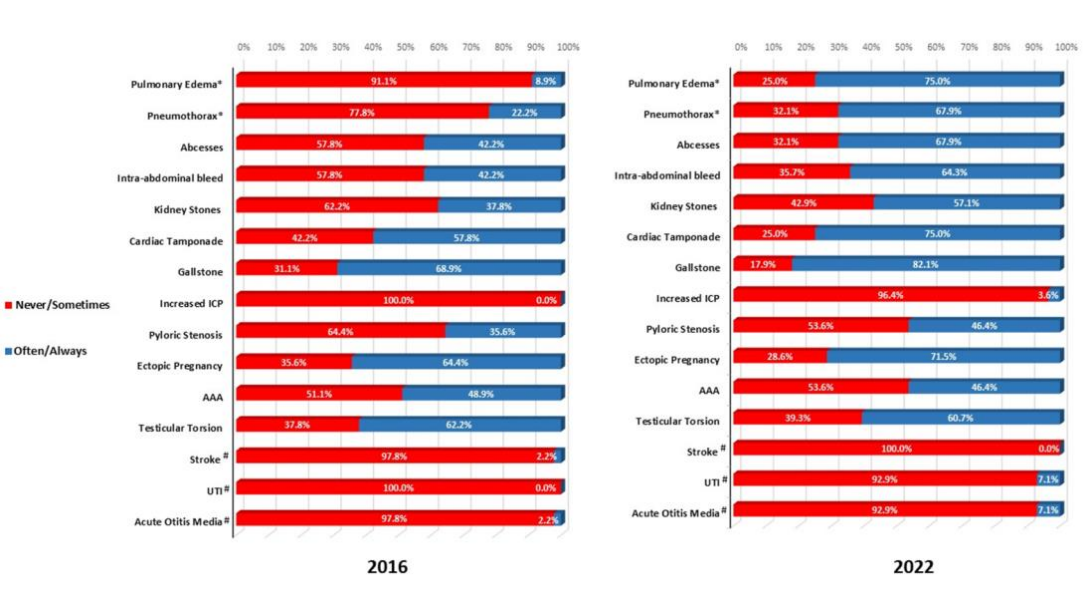
Knowledge of appropriate and inappropriate indications for POCUS, scored as the frequency with which respondents believe a clinician can use POCUS to evaluate symptoms. * = Significant p-value; # = False indication; ICP = Intracranial pressure; AAA = Abdominal aortic aneurysm; UTI = Urinary tract infection

Knowledge about the utility of POCUS in the diagnostic evaluation varied significantly by condition. Respondents were more familiar with POCUS use in conditions such as cardiac tamponade, gallstones, and ectopic pregnancy, and less aware of the role of POCUS in conditions such as elevated intracranial pressure and pyloric stenosis (Figure 1).

Overall, for positive indications, those who correctly rated a role for POCUS as “often” or “always” increased in average from 40.9% in 2016 to 59.8% in 2022. For the negative indications, those who rated a role for POCUS as “rare” or “sometimes” slightly decreased from 98.5% to 95.2%. We noted significant improvement in identifying the role of POCUS in diagnosis of pulmonary edema and pneumothorax. In 2016, only 8.9% of respondents thought there was “often” or “always” a role for POCUS in diagnosing pulmonary edema, versus 75% of respondents with similar opinions in the 2022 cohort (p<0.0001). Additionally, in 2016, 22.2% of respondents thought there was “often” or “always" a role for POCUS in diagnosing pneumothorax, versus 67.9% of respondents with similar opinions in the 2022 cohort (p=0.0002). The knowledge of the role of POCUS in diagnosing increased intracranial pressure (ICP) remained very limited with greater than 95% of respondents indicating the role of using POCUS for this diagnosis as “never” or “sometimes.”. Responses to false indications of POCUS (stroke, urinary tract infection, and acute otitis media) showed that clinical educators are aware of the lack of a role for POCUS in diagnosing of these conditions. More than 95% of respondents rated role of using POCUS for negative indications as “never” or “sometimes” in both study timepoints.

### Paired Responses

Seven paired responses were identified through the clinical educator’s semi-unique identifier, specialty, and years since residency. Six clinical educators (85.7%) had clinical teaching roles in 2016 that they all continued to perform in 2022, and the remaining clinical educator took on a clinical teaching role in 2022. Only one clinical educator was a clerkship director in 2016 and continued to have this role in 2022. One of the clinical educators was a site director in 2016 and did not continue to have this role in 2022. The majority of clinical educators did not teach didactics in either timepoint (5 and 4 clinical educators in 2016 and 2022, respectively).

In these paired responses, comfort level using or interpreting POCUS remained unchanged (p=0.543 and p=0.472, respectively). Similarly, frequency of using POCUS remained the same (p=0.548).

### Barriers to more frequent use of POCUS 

In 2022, one additional question set was included in the survey which aimed to interrogate clinical educator perspectives on specific needs to allow more frequent POCUS use (Table 3). Real-time supervision and continuing medical education (CME) were the most highly rated needs, with an average of 6.6 ± 3.2 and 6.0 ± 3.5 points on a scale of 0 to 10, respectively. However, all other needs were highly rated, including access to handheld ultrasound (5.0 ± 3.9), access to imaging archive system (5.0 ± 3.7), and protected time (4.8 ± 3.4).

**Table 3:** Barriers to more frequent use of POCUS.

**Questions** **Mean ± SD**	**2022 Respondents** **(n = 28)**
How much more likely would you be to perform POCUS yourself if you had access to:	
· A handheld or easily accessible cart-based ultrasound device	5.0 ± 3.9
· A day/weekend CME-eligible workshop on basic ultrasound skills	6.0 ± 3.5
· Real-time expert supervision to assist with acquiring and interpreting images	6.6 ± 3.2
· An image archive system for delayed expert review, feedback, and assistance with interpretation	5.0 ± 3.7
· Protected time and/or longer visits to incorporate ultrasound	4.8 ± 3.4
*On a scale of 0 to 10	

### Utility of POCUS in Primary Care Specialties 

A subset analysis for primary care specialties (Internal Medicine and Family Medicine) revealed no differences in comfort performing POCUS ratings between 2016 and 2022 (p=0.123). Around 3 in 4 (78%) clinical educators in 2016 and 1 in 2 (50%) clinical educators in 2022 were not comfortable performing POCUS. Only 2.6% in 2016 and 4.5% in 2022 were extremely comfortable performing POCUS. Despite this finding, frequency of POCUS use improved between the study timepoints. Those who never used POCUS decreased from 73.7% in 2016 to 36.6% in 2022; those who performed POCUS several times a month increased from 7.9% in 2016 to 18.2% in 2022; and those who performed POCUS several times a week increased from 2.6% in 2016 to 9.1% in 2022 (p=0.042).

Primary care specialists rated comfort in interpreting POCUS slightly better in 2022 compared to 2016, although this was not statistically significant (p=0.094). While around 3 in 4 (77.8%) were not comfortable interpreting POCUS, and 1 in 10 (10.5%) were somewhat and moderately comfortable interpreting POCUS in 2016, no clinical educator rated their comfort level interpreting POCUS as extremely comfortable. In 2022, 1 in 2 clinical educators (50.0%) were not comfortable interpreting POCUS, while (18.2%) and (27.3%) were somewhat and moderately comfortable interpreting POCUS, respectively. Only one clinical educator rated their comfort level interpreting POCUS as extremely comfortable in 2022. 

## Discussion

Multiple surveys have interrogated the extent of POCUS use and education in medical training programs, but this survey represents a unique focus on medical student clerkship-level educators over a multi-year period. 

Although the overall distribution in level of comfort did not change in a statistically significant way from 2016 to 2022, we noted a reduction in educators who are uncomfortable performing POCUS by 31.4%. Many educators gained some level of comfort with POCUS, but ultimately almost none had become “extremely comfortable” performing their own ultrasound exam. In fact, more educators described their comfort level as “somewhat” or “moderately comfortable.” Comfort level interpreting POCUS did change significantly from 2016 to 2022, suggesting that educators may have had more exposure to cognitive concepts of POCUS, with an ability to interpret basic images, even if they had not benefitted from hands-on scanning experience. Finally, the frequency of use of POCUS has significantly increased. Overall, the trends noted are indicative of growing use of POCUS with perhaps gradual improvement in comfort level performing it.

A subset analysis of responses by primary care providers alone, excluding the surgical educators, showed a more muted increase in comfort and frequency of POCUS use than the overall trends. Though the absolute numbers of surgical educators were small, this hypothesis-generating observation could suggest that increasing frequency of POCUS use might be driven in part by increasing use of POCUS in surgical and perioperative services 9.

There was noticeable discrepancies in awareness of POCUS utility for diagnostic evaluation of various clinical scenarios. Our findings indicated higher awareness for POCUS in commonly managed medical problems such as pneumothorax and pulmonary edema. For instance, the role of POCUS in pulmonary edema has been frequently discussed in recent literature resulting in increased awareness 10, 11, 12. Although there is Emergency Medicine literature regarding the use of optic nerve sheath diameter to detect findings of increased intracranial pressure 13, this is a less common indication outside of the Emergency Medicine setting and there was relatively lower awareness of the role of POCUS in this application. These trends might suggest that educators tend to focus on more frequently performed ultrasounds. Moreover, it may suggest that certain ultrasound exams are more commonly taught due to the seriousness of the illness or the feasibility of teaching the topic.

Thus, while educational programs should aim to cover a wider range of POCUS uses, the utilization of the modality may continue to vary with exposure, knowledge and experience.

Though the number of paired responses is limited, this subgroup did not appear to have significant changes in ultrasound comfort, frequency of use, or knowledge over the captured six-year period. Rather, the global changes in ultrasound use are possibly driven by hiring and promoting junior faculty who received more POCUS instruction in their own training. Multiple surveys have captured the changing landscape of ultrasound curricula in undergraduate and graduate medical education 14, 15, 16, 17, 18. For example, in one 2012 national survey, 62% of responding medical schools reported integrating ultrasound training into their curricula, and only 19% responded that it was a priority at their institution 14. It is plausible that clerkship educators who trained in this environment would not feel comfortable with POCUS use and that hiring new faculty would more effectively change this dynamic than training these established faculty through continuing medical education. More rapid uptake of this new technology has almost certainly been aided by concurrent improvements in machine portability, integration with electronic health records, and a growing evidence base supporting its use.

To inform future POCUS initiatives, we surveyed respondents about barriers to more frequent POCUS use, drawing from discussions with POCUS experts and other studies on POCUS use and including portable equipment availability, further training, real-time support for image acquisition and interpretation, delayed support for image interpretation, and supported clinical time. These were all frequently cited, and highlighted that barriers to more widespread POCUS use are multifactorial. Device access, training, support during image acquisition and interpretation, and thoughtful scheduling should all be considered in any structured intervention. Simply purchasing a handheld ultrasound or funding faculty attendance at a one-time POCUS training course cannot reasonably be expected to meaningfully change future ultrasound use.

### Limitations

This study may be limited by response rates of 46% and 31% and an overall low sample size that would be underpowered to detect subtle changes over time. However, the collected responses do capture the faculty who have a significant impact on medical student education and curricula. Among the seven internal medicine faculty responses in 2022, for example, were the one clerkship director and five other site directors. These six faculty have an outsized impact on the educational content and curricular objectives for medical students in this clerkship.

It is possible that a selection bias exists, and respondents were more likely to be enthusiastic about POCUS than non-responders. These results may therefore overestimate POCUS use and knowledge among the broader population of educators at our institution and may not be generalizable to other training environments where the medical school POCUS curriculum is more or less developed. As these biases would presumably affect both time points, the temporal trend is nevertheless instructive.

Self-reported comfort and this assessment of knowledge through POCUS indications may also not reflect individual clinical practice or POCUS competence. Adult outpatient internal medicine clinical educators may never encounter pediatric conditions such as pyloric stenosis, or emergent conditions such as tamponade. These specific diagnoses formed a small portion of the overall content assessed.

## Conclusions

We have shown that over a six-year period at one urban, academic medical center, comfort with POCUS and frequency of use have increased slightly but remain low among core faculty responsible for medical student clerkship education. There are still large gaps in knowledge and very few faculty regularly use POCUS, which can be attributed to a variety of barriers including device access, training, and expert support. Future initiatives for POCUS in undergraduate medical education should take these factors into account to better support the educators who are designing and delivering curricula.

## Conflict of Interests

Authors declare no conflict of interests relevant to this work.

## Funding

None.

## Authorship Statement

NS, WC, CB, and NP contributed to the conception and design of the research. NS contributed to the acquisition of data. FS, NS, and MME contributed to analysis of data. NS, MME, and NP contributed to interpretation of data. MME and NS drafted the manuscript. All authors critically revised the manuscript, agree to be fully accountable for ensuring the integrity and accuracy of the work, and read and approved the final manuscript. 
